# Rate of Force Development as an Indicator of Neuromuscular Fatigue: A Scoping Review

**DOI:** 10.3389/fnhum.2021.701916

**Published:** 2021-07-09

**Authors:** Samuel D’Emanuele, Nicola A. Maffiuletti, Cantor Tarperi, Alberto Rainoldi, Federico Schena, Gennaro Boccia

**Affiliations:** ^1^Department of Neurosciences, Biomedicine and Movement Sciences, University of Verona, Verona, Italy; ^2^Human Performance Lab, Schulthess Clinic, Zurich, Switzerland; ^3^Department of Clinical and Biological Sciences, University of Turin, Turin, Italy; ^4^Department of Medical Sciences, University of Turin, Turin, Italy; ^5^NeuroMuscularFunction | Research Group, School of Exercise and Sport Sciences (SUISM), University of Turin, Turin, Italy; ^6^CeRiSM, Sport Mountain and Health Research Centre, University of Verona, Verona, Italy

**Keywords:** contraction quickness, force-time curve, knee extension, explosiveness, fatigability

## Abstract

Because rate of force development (RFD) is an emerging outcome measure for the assessment of neuromuscular function in unfatigued conditions, and it represents a valid alternative/complement to the classical evaluation of pure maximal strength, this scoping review aimed to map the available evidence regarding RFD as an indicator of neuromuscular fatigue. Thus, following a general overview of the main studies published on this topic, we arbitrarily compared the amount of neuromuscular fatigue between the “gold standard” measure (maximal voluntary force, MVF) and peak, early (≤100 ms) and late (>100 ms) RFD. Seventy full-text articles were included in the review. The most-common fatiguing exercises were resistance exercises (37% of the studies), endurance exercises/locomotor activities (23%), isokinetic contractions (17%), and simulated/real sport situations (13%). The most widely tested tasks were knee extension (60%) and plantar flexion (10%). The reason (i.e., rationale) for evaluating RFD was lacking in 36% of the studies. On average, the amount of fatigue for MVF (−19%) was comparable to late RFD (−19%) but lower compared to both peak RFD (−25%) and early RFD (−23%). Even if the rationale for evaluating RFD in the fatigued state was often lacking and the specificity between test task and fatiguing exercise characteristics was not always respected in the included studies, RFD seems to be a valid indicator of neuromuscular fatigue. Based on our arbitrary analyses, peak RFD and early phase RFD appear even to be more sensitive to quantify neuromuscular fatigue than MVF and late phase RFD.

## Introduction

The magnitude of neuromuscular fatigue—also referred to as muscle fatigue (Gandevia, 2001) or neuromuscular fatigability (Chartogne et al., 2020)– is universally evaluated as the exercise-induced decline in the isometric maximal voluntary contraction force (hereafter abbreviated as MVF) of a muscle/muscle group. In this context, pre- to post-fatigue percent declines in knee extension MVF ranging from 8 to 34% have been reported for a multitude of exercise types of different duration and intensity (Millet and Lepers, 2004). Nevertheless, the validity of this approach/variable can partially be questioned as the characteristics of the fatiguing exercise (e.g., explosive jumps) do not always correspond to those of the testing contraction/task (e.g., slow ramp and hold knee extension). This lack of task specificity may result in an underestimation of the magnitude of neuromuscular fatigue, thereby suggesting the need for evaluating outcome measures other than the classical MVF.

The rate of force development (RFD)—which is basically obtained from the ascending part of the force-time curve of an explosive contraction either as a mean time-locked value or a maximal force per time ratio—has received increasing interest in the last few years for the evaluation of explosive strength in multiple situations (Maffiuletti et al., 2016; Rodriguez-Rosell et al., 2017). As such, RFD has been shown to be more sensitive than MVF to detect chronic changes induced for example by aging (Thompson et al., 2014), immobilization/disuse (de Boer et al., 2007), strength training (Andersen et al., 2010) and rehabilitation (Angelozzi et al., 2012), but also acute adjustments associated to exercise (Buckthorpe et al., 2014), muscle damage (Peñailillo et al., 2015), and pain (Rice et al., 2019). Despite being more functionally relevant than pure maximal strength (Tillin et al., 2010; McLellan et al., 2011), RFD—particularly the one derived from the earlier phase of the contraction (≤100 ms; early RFD)—has been suggested to be largely influenced by neural mechanisms, mainly in relation with motor unit behavior (Del Vecchio et al., 2019). This unique physiological feature of RFD could explain, at least in part, why this variable has often been found to be more sensitive to changes than MVF.

Although the effect of neuromuscular fatigue on maximal strength was documented 130 years ago by Angelo Mosso (1891), the impact of fatigue on the ascending part of the force-time curve was described only recently. Royce (1964) was the first to report a similar fatigue-related decline in MVF (47%) and peak RFD (50%) after a sustained (1 min) maximal contraction of the finger flexors. Later, Viitasalo and Komi (1981) found that 100 explosive contractions of the knee extensor muscles decreased MVF and peak RFD, respectively, by 24 and 36%. In the same year, Kearney and Stull (1981) investigated RFD across many non-overlapping time intervals, and reported that early RFD was more affected than late RFD (>100 ms) following a sustained maximal contraction of the finger flexor muscles. Since these seminal reports, numerous studies have been published on the fatigue-related changes in RFD of different muscle groups and for different types of exercises, including actual sport situations. Nevertheless, a comprehensive understanding of the effect of neuromuscular fatigue on RFD—and more particularly so in relation with MVF—is still lacking.

Because RFD represents a valid alternative/complement to the classical evaluation of pure maximal strength in unfatigued conditions (Maffiuletti et al., 2016), the aim of this scoping review was to map the available evidence regarding RFD as a possible indicator of neuromuscular fatigue. Thus, following a general overview of the different studies published on this topic, we formulated two main research questions. The primary question was: “Is RFD a valid indicator of neuromuscular fatigue?” To address this question we arbitrarily compared the magnitude of neuromuscular fatigue—characterized by the exercise-induced decline in selected variables—between MVF (“gold standard”) and peak RFD (i.e., the most commonly evaluated RFD variable). The secondary research question of this study was: “What is the most sensitive RFD variable for evaluating neuromuscular fatigue?” To address this question we arbitrarily compared the magnitude of neuromuscular fatigue between different RFD variables—basically peak, early and late RFD—always in relation with MVF.

## Methods

### Protocol and Eligibility Criteria

The protocol was drafted using the Preferred Reporting Items for Systematic Reviews and Meta-analysis Protocols for Scoping Review (PRISMA-ScR) (Tricco et al., 2018). A literature search was conducted in February 2020 on PubMed, Scopus, and Web of Science databases. Peer-reviewed journal articles in English were included if: (1) the study involved healthy human participants, (2) at least one key term of the search string (see below) was included within the title, abstract, or keywords, (3) voluntary contractions were used to evaluate RFD before (pre-test) and within 1 h after the end of a standardized fatiguing exercise (post-test). The exclusion criteria were: (1) reviews, (2) studies whose main focus was not neuromuscular fatigue (e.g., post-activation potentiation), (3) studies in which fatigue was induced by non-voluntary contractions, (4) studies in which RFD was evaluated during vertical jumps due to the impossible comparison with MVF, (5) studies with missing data not obtained even after having contacted the corresponding author by e-mail. If the study design included the ingestion of dietary supplements, only the control group was considered.

### Search Strategy

A Boolean search strategy was applied using the following string: (“rate of force development” OR “rate of torque development” OR “explosive contraction” OR “ballistic contraction” OR “time to peak force” OR “time to peak torque” OR “time to maximal force” OR “rate of force production” OR “force pulses” OR “force impulses” OR “torque pulses” OR “torque impulses” OR “rapid contraction”) AND (“fatigue” OR “fatiguing” OR “fatigability”).

### Selection of Sources of Evidence

The final search results were exported into EndNote^1^, and duplicates were removed. Further on, the reference list was imported into Rayyan^2^ and abstracts were evaluated independently by two authors (SD’E and GB), in a blinded mode. At last, all selected articles were read. Corresponding authors of the selected articles were eventually contacted by e-mail to request any missing relevant information.

### Data Items

We extracted the following data from the included articles: (1) subject characteristics (sample size, gender, age group, sport/training status); (2) fatiguing exercise characteristics; (3) test task characteristics; (4) percent decline (pre- to post-test) for four variables of interest: MVF, peak RFD, early RFD (≤100 ms), and late RFD (>100 ms). If there was more than one estimate for early and late RFD (e.g., RFD 0–50 and 0–100 ms for early RFD) or more than one arm for each study, we averaged the values for each variable of interest. If percent declines of MVF and RFD were not available, they were calculated from absolute data using the following formula: $\%=\frac{\left(\mathrm{post}-\mathrm{pre}\right)}{\text{pre}}\cdot 100.$

### Arbitrary Synthesis of the Data

We arbitrarily clustered the fatiguing exercises in two groups: “strength exercises,” including isometric and isokinetic contractions, vertical jumps, resistance/strength exercises, etc., and “other exercises” including endurance exercises/locomotor activities (e.g., running, cycling, swimming), simulated/real sport situations (e.g., half-marathon, soccer match, handball training session) and combined (strength and endurance) exercises. To arbitrarily synthesize the results, we averaged the percent declines of MVF, peak RFD, early and late RFD by fatiguing exercise type (strength vs. other exercises). The primary research question (concurrent validity of RFD) was addressed by comparing the percent decline of MVF to peak RFD. The secondary research question (sensitivity of RFD to changes induced by exercise) was addressed by comparing percent declines between the different RFD variables. Then, we also categorized studies based on whether the test task corresponded or not to the fatiguing task. When fatigue was evaluated with the same task adopted to induce fatigue (e.g., knee extension)—irrespective of the action mode—the test was considered “specific,” otherwise it was considered “non-specific.” Finally, we also verified if the reason (i.e., the scientific rationale) for evaluating RFD was provided in the introduction of all selected studies.

## Results

### Selection of Sources of Evidence

**FIGURE 1:**
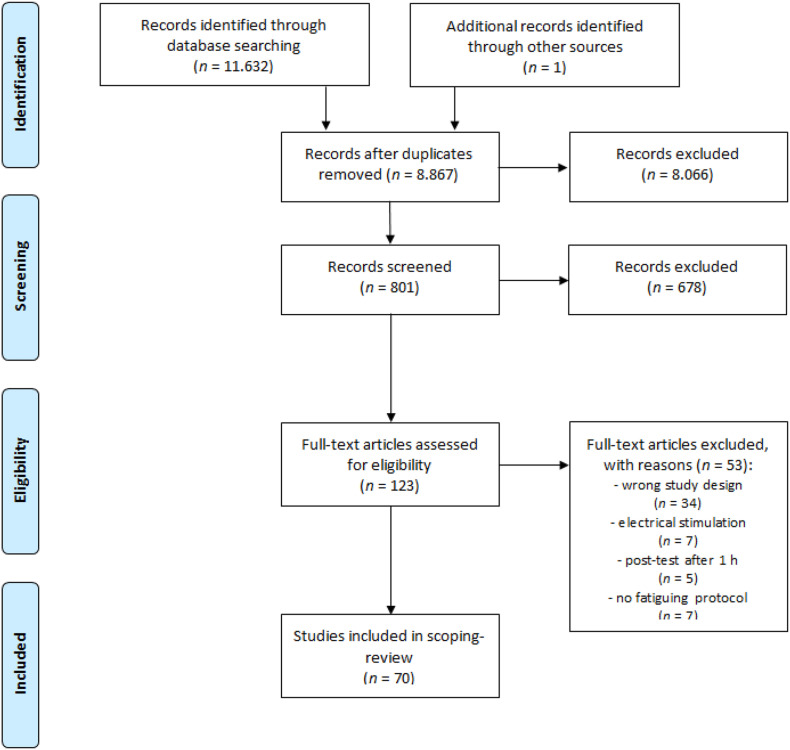
PRISMA flow diagram.

The electronic database search resulted in the identification of 8,867 potential studies after duplicate removal (Figure 1). Following a preliminary inspection of title, abstract and keywords, 8,066 articles were excluded and 801 studies were available for screening. Through accurate examination of the abstracts, 678 studies were excluded and 123 studies were assessed for eligibility. Based on the exclusion criteria, 53 studies were excluded and 70 full-text articles were ultimately included in the review.

### Results of Individual Sources of Evidence

The characteristics of the subjects, fatiguing exercise and test task, as well as the pre- to post-test percent declines for MVF, peak RFD, early RFD and late RFD are presented in Tables 1, 2 for strength exercises (43 studies) and other exercises (27 studies), respectively. Publication dates ranged from 1981 to 2020 with a notable increment starting from 2012. Only 14.5% of the total number of subjects (*n* = 1,206) were women. The training status of the participants was distributed as follows: physically active (56%), elite/professional athletes (11%), inactive (7%), or not declared (26%). Age groups were distributed as follows: adults (51%), young adults (43%), old (4%), middle-aged adults (1%), and children (1%). The fatiguing exercise was conducted in laboratory conditions in 59 studies (84%) and on the field in 11 studies (16%). The most-common fatiguing exercises consisted of resistance exercises (37%), endurance exercises/locomotor activities (23%), isokinetic contractions (17%), simulated/real sport situations (13%), and vertical jumps (6%). The most widely tested task was knee extension (42 studies, 60%), followed by plantar flexion (7 studies, 10%), and knee flexion (6 studies, 9%). RFD was tested using isometric contractions in 67 studies (96%), and with dynamic actions in only three studies (4%). The rationale for evaluating RFD was lacking in 25 studies (36%), this variable not even being mentioned in the study aims.

**TABLE 1:** Study characteristics and pre-to-post fatigue percent declines of selected variables induced by “strength” exercises.

**References**	**Subjects**	**Fatiguing exercise**	**Test task**	**MVF decline**	**Peak RFD decline**	**Early RFD decline**	**Late RFD decline**
Alhammoud et al. (2018)	22♂ elite alpine skiers	35 max isokinetic KE	KE	−20%		−5%^§^	−5%^§^
Andersen et al. (2014)	20♀	100 max isometric SE	SE	↓	↓		
Balshaw et al. (2017)	10♂ resistance-trained	4 × 3 × 3 concentric-eccentric KE protocols ≠ loads and durations	KE	−4%^§^		−2%^§^	−2%^§^
Battazza et al. (2019)	20♂	10 × 8 concentric KE-KF	KE	−55%	−76%		
Behrens et al. (2012)	8♂ and 7♀	4 × 25 max concentric-eccentric	KE	−40%	−40%		
Brandon et al. (2015)	10♂ elite strength and power athletes	10 × 5 50–85% max SQT	KE	−8%^§^	−17%^§^		
Buckthorpe et al. (2014)	11♂ untrained	10 × 5 (3 s) explosive KE	KE	−42%	−26%	−29%	
Cadore et al. (2013)	11♂ national rugby players	100/200/300 hurdle jumps	KE	−12%^§^	−13%^§^		
Cadore et al. (2018)	14♀ and 8♂ active	4 × 20 max concentric or eccentric contractions KF-KE	KE	−24%^§^	−40%^§^		
Cerqueira et al. (2019)	13♂ active/very active	Time to fatigue intermittent isometric HG 45% max	HG			−44%^§^	−52%
Conchola et al. (2015)	17♂ resistance-trained	5 × 8 80% max or 5 × 16 40% max SQT	KE	−18%^§^	−29%^§^	−27%^§^	−21%^§^
Drake et al. (2019)	8♂ and 2♀ resistance-trained	8 × 3 SQT	SQT	−9%^§^	−9%^§^	−1%^§^	−8%^§^
Ereline et al. (2004)	11♂ powerlifters and 14♂ untrained	30 max concentric isokinetic KE	KE	↓			↓
Ewing and Stull (1984)	28♀	Time to fatigue HG 40, 60 and 80%max	HG	−31%^§^		−30%^§^	−36%^§^
Farney et al. (2018)	11♂ trained	3× barbell thrusters + jump SQT + lunge jumps + forward jumps	MTP	−5%	−4%		
Gordon et al. (2017)	9♂ and 10♂ recreationally active middle-aged adults	8 × 10 concentric KE-eccentric KF	KE	−29%^§^			−37%^§^
Häkkinen and Myllylä (1990)	33♂ active or endurance or resistance-trained	Time to exhaustion bilateral KE 60% max	KE	−26%	−33%		
Hatzikotoulas et al. (2014)	10♂ children and 11♂ active	Time to exhaustion max PF	PF	−49%^§^	↓^§^		
Jenkins et al. (2014)	18♂	6 × 10 max eccentric isokinetic FF	FF	−47%	−55%	−60%^§^	−52%
Kearney and Stull (1981)	15♂	Time to fatigue HG 40, 60, 80% max	HG	−40%^§^		−53%^§^	−29%^§^
King et al. (2012)	13♂ and 12♂ non-sedentary young olds	Max isokinetic PF	PF	−50%^§^	−10%^§^		
Linnamo et al. (1998)	8♂ and 8♀ physically fit	5 × 10 and 5 × 10 40% max KE	KE	−17%^§^	−21%^§^		
Marshall et al. (2012)	14♂ resistance trained	5 × 4; 5 × 4, with 20 s inter-set rest intervals SQT	SQT	−8%^§^	−11%^§^		
Marshall et al. (2015)	10♂ resistance trained	2 × 30 s KE 80% max + time to exhaustion and 2 × 60 KE 40% max + time to exhaustion	KE	↓^§^	↓^§^	↓^§^	
Marshall et al. (2018)	8♂ resistance trained	Full-body resistance-training 3–4 sets × 4–12 reps	KE	−28%	NS	NS^§^	
Marshall et al. (2020)	8♂ and 8♀ resistance trained	Full-body resistance-training 4 × 6 ≠ intensities	KE	−11%	NS		
McCaulley et al. (2009)	10♂ strength trained	SQT 4 × 10 75% max or 11 × 3 90% max or 8× 6 jumps	SQT	↓	↓		
Metcalf et al. (2019)	8♂ and 8♀ resistance trained	KE from 60 to 90% max + time to exhaustion 80% max	KE	−26%^§^	↓^§^	↓^§^	
Minshull and James (2013)	10♂ recreationally active	3 × 30 s max KE	KE	−12%		−21%	
Moreira et al. (2015)	19♂ professional soccer players	Time to fatigue max isokinetic concentric alternated KE and KF	KE	−24%		−25%^§^	−24%
			KF	−24%		−38%^§^	−35%
Morel et al. (2015)	11♂ well trained	20 × 8 max isokinetic KE	KE	+33%		−45%	
Nicholson et al. (2014)	7♂ resistance-trained	4 × 6 85% max and 4 × 10 70% max SQT	SQT	−16%^§^		−22%^§^	−21%
Orssatto et al. (2018)	7♀ and 15♂ young olds	LP and KF 60 and 85% max	KE	−16%^§^		−26%^§^	−19%^§^
Patrizio et al. (2018)	10♂ trained	Full-body resistance-training exercises 3 × 8 80% max	KE	−9%			−8%
Power et al. (2013)	8♂ and 8♀ recreationally active	5 × 30 eccentric isokinetic DF	DF	−28%^§^	−22%^§^		
Storey et al. (2012)	13♂ and 3♀ weightlifters and 13♂ and 3♀ resistance-trained	10 front SQT 90% max	SQT	−16%^§^	NS^§^	NS^§^	NS^§^
Strojnik and Komi (2000)	12♂ active	Time to exhaustion sledge jumps 60% max height	KE	−18%	−38%		
Váczi et al. (2013)	8♂ active	Two protocols: 10 × 10 one-leg stair-jump and level jump	KE	−7%^§^		−25%^§^	
Valkeinen et al. (2002)	29♂ and 28♀ inactive or moderately active	Time to exhaustion NE and NF 60% max	NE	−15%^§^	−17%^§^		
			NF				
Vila-Chã et al. (2012)	10♂ adults	4 × 25 max eccentric KE	KE	−14%	−14%		
Wallace et al. (2016)	10♂ recreationally active and 10♂ active olds	2 × 25 PF 20% max	PF	−23%^§^	−37%^§^		
Zhou (1996)	7♂ and 4♀ phys. education students	25 isometric KE max	KE	−57%	−56%		
Zhou et al. (1998)	4♂ and 3♀	25 isometric KE max	KE	−55%	−53%		
**Grand mean**				**−23%**	**−30%**	**−28%**	**−25%**

*For each study, empty boxes indicate that data were not measured/reported. If not otherwise specified, subjects are adults. DF, dorsiflexion; FF, forearm flexion; HG, handgrip; KE, knee extension; KF, knee flexion; MTP, mid-thigh pull; MVF, maximal voluntary force; NE, neck extension; NF, neck flexion; NS, not significant; PF, plantar flexion; RFD, rate of force development; SE, shoulder elevation; SQT, squat;↓ or ↑, the authors reported a decrease or increase without reporting an unequivocal value; §, mean of values when merging arms of the study.*

**TABLE 2:** Study characteristics and pre-to-post fatigue percent declines of selected variables induced by “other” exercises (endurance, locomotor, sport, combined).

**References**	**Subjects**	**Fatiguing exercise**	**Test task**	**MVF decline**	**Peak RFD decline**	**Early RFD decline**	**Late RFD decline**
Bassan et al. (2016)	15♂ swimmers	Time to exhaustion swimming	EF	−16%	−18%		
			EE	−10%	−9%		
Boccia et al. (2017a)	14♂ amateur runners	Half-marathon	KE	−22%	−24%	−33%^§^	−27%
Boccia et al. (2017b)	16♂ well trained XC skiers	56 km cross-country skiing	KE	−13%	−11%	−18%^§^	−10%^§^
			EE	−6%	−26%	−22%^§^	−8%^§^
Boccia et al. (2018a)	23♂ runners	Half-marathon	KE	−21%	−19%		
Boccia et al. (2018b)	11♂ and 10♀ amateur runners	Half-marathon	KE	−11%^§^	−15%^§^		
Conceição et al. (2014)	13♂ active	6 × 8 75% max squat or 6 × 8 counter movement jumps + cycling time to exhaustion 2nd ventilatory threshold	LL	−16%^§^	−21%^§^		
Dorneles et al. (2020)	22♂ and 2♀	30 min walking	HF	−4%		−15%^§^	−11%
Girard et al. (2013)	12♂ active	10 × 6 s all out + 5 × 6 s cycling sprints	KE	−12%		−29%^§^	−16%
Girard et al. (2014)	12♂ tennis players	≈2 h tennis in hot and cool condition	KE	−16%^§^	−16%^§^		
			PF	−12%^§^	−1%^§^		
Girard et al. (2015)	17♂ elite soccer players	Soccer match in hot and cool condition	PF	−6%^§^	−13%^§^		
Girard et al. (2016)	13♂ recreational team sport athletes	8 × 5 s all-out run sprints	KE	−9%	−5%	−8%	−10%
Grazioli et al. (2019)	16♂ professional soccer players	Soccer match	KE	0%		+22%	+3%
			KF	−1%		−16%	−11%
Greco et al. (2013)	22♂ professional soccer players	Soccer specific intermittent protocol	KE	−14%		−14%^§^	
			KF	−18%		−17%^§^	
Kelly et al. (2011)	12♂ recreational athletes	1 h running at 1st ventilatory threshold + 10%	PF	−17%			−17%
Krüger et al. (2019)	10♂ physically active	Three cycling protocols: 30 s all-out/10 min severe/90 min moderate intensity	LL	−26%^§^	↓^§^		
Lapole et al. (2013)	10♂ volleyball players	10 min volleyball specific circuit	PF	−12%	−18%		
Marshall et al. (2014)	8♂ amateur soccer players	Soccer-specific aerobic field test	KF	−24%	−31%	−67%^§^	
Oliveira et al. (2013)	8♂ physically active	≈35 min running 95% onset blood lactate accumulation	KE	−4%		−15%	
Peñailillo et al. (2015)	10♂	30 min eccentric cycling 60% peak power	KE	−19%			−23%
Alota Ignacio Pereira et al. (2018)	119♂ from young adults to olds	30 min sit-to-stand or time to exhaustion	LL	−11%^§^		−14%^§^	−2%^§^
Ravier et al. (2018)	9♂ professional handball players	Handball specific circuit	KE	−19%		−24%	
Rissanen et al. (2020)	27♂ active	Three different protocols with resistance exercise or cycling or combined	LP	−16%^§^	−27%^§^		
Siegler et al. (2013)	8♂ and 2♀ active	Time to exhaustion cycling 120% peak power for 30 s with 30 s recovery	LL	−27%	−32%	−55%^§^	−29%
Taipale and Häkkinen (2013)	12♂ and 10♀ recreational runners	45 min resistance-training circuit + 60 min steady-state running or vice versa	LP	−16%^§^	−19%^§^		
Thorlund et al. (2008)	10♂ elite handball players	Simulated handball match	KE	−11%	−21%	−17%^§^	−16%
			KF	−10%	−21%	−2%^§^	−17%
Thorlund et al. (2009)	9♂ soccer players	Soccer match	KE	−11%		−7%	−8%
			KF	−7%		−7%	−9%
Zhou et al. (1996)	6♂ untrained	Cycling 4 × 30 s all out	KE	−49%	−62%		
**Grand mean**				**−14%**	**−20%**	**−19%**	**−13%**

*For each study, empty boxes indicate that data were not measured/reported. If not otherwise specified, subjects are adults. EE, elbow extension; EF, elbow flexion; HF, hip flexion; KE, knee extension; KF, knee flexion; LL, lower limb; LP, leg press; MVF, maximal voluntary force; PF, plantar flexion; RFD, rate of force development; ↓ or ↑, the authors reported a decrease or increase without reporting an unequivocal value; §, mean of values when merging arms of the study.*

### Arbitrary Synthesis of Results

On average, the mean percent decline of peak RFD (−25%) was 6% larger compared to MVF (−19%), and this result was observed in 28 out of 41 studies. The greater decline of peak RFD against MVF was consistently observed for both strength exercises (MVF: −23%, peak RFD: −30%) and other exercises (MVF: −14%, peak RFD: −20%). Similarly, the mean percent decline of early RFD (−23%) was 4% larger compared to late RFD (−19%), this result being observed in 13 out of 21 studies and for both strength and other exercises. Of note, percent declines were similar for late RFD and MVF (both −19%), as well as for early RFD and peak RFD (−23 and −25%, respectively).

When fatigue was evaluated with the same task adopted to induce fatigue (50% of the studies), both MVF and peak RFD percent declines (MVF: −27%, peak RFD: −33%) were larger compared to when fatigue was evaluated with a non-specific task (MVF: −15%, peak RFD: −23%), e.g., when knee extension was used to quantify fatigue induced by a locomotor exercise.

## Discussion

In this scoping review, we identified 70 primary studies addressing the acute effect of different types of fatiguing exercises on RFD. Our findings indicate that the classical exercise-induced alteration in MVF was typically accompanied by a decline in RFD, this latter being markedly affected by neuromuscular fatigue. Early phase RFD and peak RFD appeared to be more sensitive to detect neuromuscular fatigue than MVF and late phase RFD. However, the rationale for evaluating RFD in the fatigued state was often lacking and the specificity between test task and fatiguing exercise characteristics was not always respected in the included studies.

### Peak RFD vs. MVF

In the presence of an exercise-induced decrease in maximal strength (MVF), an impairment in explosive strength (RFD) should also be expected as the force-time curve is typically shifted to the right under fatigued conditions (Valkeinen et al., 2002). Consequently, it takes longer to produce the same amount of force and the force available at a given time point is lower. Peak RFD, which is calculated as the highest rate of force increase along the force-time curve (steepest slope), is usually the most investigated RFD variable (see also Tables 1, 2). On average, peak RFD decreased more than MVF (−25 and −19%, respectively), and most studies reported, at least in one arm of their experiments, a larger decline in peak RFD than in MVF. Therefore, peak RFD was more susceptible to exercise-induced neuromuscular fatigue compared to MVF. Interestingly, it was true even if the between-session reliability of peak RFD—despite being acceptable and better than the other RFD variables—was generally lower compared to MVF (Buckthorpe et al., 2012; Haff et al., 2015). For example, Buckthorpe et al. (2012) reported intraclass correlation coefficients of 0.95, 0.90, and 0.80 for knee extension MVF, peak RFD and early RFD (0–50 ms), respectively. Nevertheless, there were also fatigue studies reporting similar or even smaller changes for peak RFD than for MVF. The large variety of experimental and methodological conditions did not allow to formulate a more specific hypothesis on which fatigue protocols may induce larger declines in RFD compared to MVF.

### Early vs. Late RFD

Different mechanisms seem to govern early and late RFD and their relative contribution may vary throughout the time course of the force-time curve rise (Cossich and Maffiuletti, 2020). Broadly speaking, early RFD is poorly correlated to MVF (Andersen and Aagaard, 2006) and is largely dependent on motor unit recruitment speed and maximal discharge rate (Del Vecchio et al., 2019). On the other hand, late RFD is strongly correlated to MVF and therefore seems to depend more on structural variables such as muscle cross-sectional area and architecture (Andersen et al., 2010; Folland et al., 2014). Therefore, analyzing RFD at different time intervals provides the framework for a more articulated understanding of the underlying mechanisms. On average, early RFD decreased more than late RFD (−23 and −19%, respectively) as a result of fatiguing exercise. This may suggest that early contraction phases may be particularly sensitive to identifying neuromuscular fatigue. Of note, the average decline in late RFD was very similar to the one of MVF. This seems to confirm that late RFD would provide similar results than MVF likely because these two variables are highly correlated and share similar physiological determinants (Andersen et al., 2010; Folland et al., 2014). Based on the current data, it can be recommended that including the analysis of early RFD would add meaningful insights to neuromuscular fatigue quantification, while late RFD may be redundant with respect to MVF.

### Peak RFD

As already discussed, the most reliable RFD variable is peak RFD (Buckthorpe et al., 2012) and it is also probably the easiest to calculate, as it only requires extracting the maximal value from the first derivative of the force signal. This may explain, for example, why Drake et al. (2019) observed a significant decline of peak RFD after a strength training session but no changes in RFD for different time intervals up to 200 ms. Such higher reliability of peak RFD may help disclosing fatigue-related differences otherwise undetected by time-locked RFD variables. On a side note, peak RFD typically occurs between 30 and 100 ms after contraction onset (Gruber and Gollhofer, 2004), this could explain why fatigue-induced changes in peak RFD are consistent with early RFD changes.

### Rationale for RFD and Methodological Considerations

Although RFD is increasingly considered as a relevant index of neuromuscular function (Maffiuletti et al., 2016; Buckthorpe and Roi, 2017), very few fatigue studies were specifically designed to measure RFD as the primary outcome and no rationale was clearly presented for its evaluation. It seems indeed quite contradictory to implement RFD assessments before and after fatiguing exercise induced by non-explosive contractions such as slow resistance exercise or walking. We rather believe that RFD should better be evaluated following fatiguing exercises based on (relatively) rapid contractions. Several methodological details regarding RFD evaluation were not provided in most of the studies included in the present review. These details (reviewed here: Maffiuletti et al., 2016) include the instructions given to participants, the time window for peak RFD quantification and the eventual length of the moving window, the method adopted to identify contraction onset, and the number of test trials (that is particularly relevant in fatigued conditions). More efforts should be made in future fatigue studies in this direction.

### RFD During Recovery

Besides the magnitude of neuromuscular fatigue, recovery time-course can also be influenced by the choice of the outcome. Four studies showed that RFD recovery was slower than MVF after exercise termination (Viitasalo and Komi, 1981; Zhou, 1996; Conchola et al., 2015; Krüger et al., 2019). For example, Zhou (1996) found that after 25 maximal voluntary contractions, MVF was restored in 10 min but peak RFD did not completely recover even after 20 min. While there were also studies showing similar recovery profiles between RFD and MVF (Linnamo et al., 1998; Marshall et al., 2012), there was an overall trend for longer-lasting exercise-induced declines of RFD as compared to MVF. If future studies would confirm this observation, it would make RFD a promising indirect marker of post-exercise recovery kinetics. As low-frequency fatigue is suspected to be one of the neuromuscular impairments lasting for longer after exercise termination (Jones, 1996), this may suggest that the more protracted RFD depression may be linked to low-frequency fatigue (Krüger et al., 2019), such as in the presence of eccentric-induced muscle damage. Interestingly, both early and late RFD have been found to be more affected than MVF following 60 eccentric contractions (Jenkins et al., 2014) and 30 min of eccentric cycling (Peñailillo et al., 2015). This would potentially indicate that RFD may be more sensitive than MVF to muscle damage induced by eccentric contractions. However, it is still unclear which time interval would be more suitable to consider.

### Test Specificity

The similarity between the test and the fatiguing exercise in terms of task and contraction characteristics is crucial. When the task adopted to quantify fatigue was similar to the task adopted to induce fatigue in the studies we considered, the decline in peak RFD was on average 33 vs. 23% when fatigue was evaluated with a non-specific task. Neuromuscular fatigue induced by locomotor activities or multi-joint resistance exercises was often quantified using single-joint tasks, such as the universally employed knee extension. This was done in the hope that the single-joint task may provide a surrogate measure of fatigue occurring in the multi-joint task. While being the easiest and fastest way to evaluate RFD, this approach inevitably minimizes the magnitude of fatigue and left rooms of unknown. In an attempt to increase the external validity of measuring RFD in fatigued conditions, we recommend that the test task should be as specific as possible to the fatiguing task, as done for example by Marshall et al. (2012) and Drake et al. (2019).

### Limitations

This review has some limitations. We did not perform a meta-analysis of percent decline data because the study design and calculating/reporting of RFD were too disparate in the included studies. Furthermore, many studies did not fully report the basic data (e.g., mean and standard deviation of pre- and post-tests), and these data were still unavailable even after having contacted the corresponding authors. When the percent decline was lacking, we calculated it based on averaged group estimates, and this may have induced inconsistencies among studies. As most studies investigated the knee extensor muscles, it is unclear if the present results may be extended with confidence to other muscle groups. As only 14.5% of participants were women, the main findings of the present review are probably more meaningful for men, even though the studies that investigated gender differences found similar percent declines between men and women (Linnamo et al., 1998; Valkeinen et al., 2002; Lanning et al., 2017; Boccia et al., 2018b; Marshall et al., 2020). Finally, due to the already-discussed heterogeneity across studies, the relative effect of age and training status could not be examined, and no attempt was made to discuss the main results in relation with the origin of neuromuscular fatigue (central vs. peripheral).

## Conclusion

We conclude by suggesting that RFD is a valid indicator of neuromuscular fatigue. More specifically, we demonstrated that peak RFD may be more susceptible to exercise-induced fatigue compared to the classical MVF, and the analysis of early RFD might provide more useful information than late RFD.

## Author Contributions

NAM, SD’E, and GB designed the study, interpreted the data, and wrote the first draft of the manuscript. SD’E organized the database and collected the data. SD’E and GB performed the analytical evaluation of articles. NAM and SD’E performed the statistical analysis. All authors contributed to manuscript revision, conceived the study, read, and approved the submitted version.

## Conflict of Interest

The authors declare that the research was conducted in the absence of any commercial or financial relationships that could be construed as a potential conflict of interest.

## Footnotes

1 https://endnote.com

2 https://rayyan.qcri.org

## References

[B1] Alhammoud M. Morel B. Girard O. Racinais S. Sevrez V. Germain A. (2018). Hypoxia and fatigue impair rapid torque development of knee extensors in elite alpine skiers. *Front. Physiol.* 9:962. 10.3389/fphys.2018.00962 30140231 PMC6094991

[B2] Alota Ignacio Pereira V. Augusto Barbieri F. Moura Zagatto A. Cezar Rocha, Dos Santos P. Simieli L. (2018). Muscle fatigue does not change the effects on lower limbs strength caused by aging and Parkinson’s disease. *Aging. Dis.* 9 988–998. 10.14336/ad.2018.0203 30574412 PMC6284767

[B3] Andersen L. L. Aagaard P. (2006). Influence of maximal muscle strength and intrinsic muscle contractile properties on contractile rate of force development. *Eur. J. Appl. Physiol.* 96 46–52. 10.1007/s00421-005-0070-z 16249918

[B4] Andersen L. L. Andersen C. H. Skotte J. H. Suetta C. Søgaard K. Saltin B. (2014). High-intensity strength training improves function of chronically painful muscles: case-control and RCT studies. *BioMed. Res. Int.* 2014:187324. 10.1155/2014/187324 PMC3953472 24707475

[B5] Andersen L. L. Andersen J. L. Zebis M. K. Aagaard P. (2010). Early and late rate of force development: differential adaptive responses to resistance training? *Scand. J. Med. Sci. Sports* 20 e162–e169. 19793220 10.1111/j.1600-0838.2009.00933.x

[B6] Angelozzi M. Madama M. Corsica C. Calvisi V. Properzi G. Mccaw S. T. (2012). Rate of force development as an adjunctive outcome measure for return-to-sport decisions after anterior cruciate ligament reconstruction. *J. Orthop. Sports Phys. Ther.* 42 772–780. 10.2519/jospt.2012.3780 22814219

[B7] Balshaw T. G. Pahar M. Chesham R. Macgregor L. J. Hunter A. M. (2017). Reduced firing rates of high threshold motor units in response to eccentric overload. *Physiol. Rep.* 5:e13111. 10.14814/phy2.13111 28108648 PMC5269413

[B8] Bassan N. M. César T. E. Denadai B. S. Greco C. C. (2016). Relationship between fatigue and changes in swim technique during an exhaustive swim exercise. *Int. J. Sports Physiol. Perform.* 11 33–39. 10.1123/ijspp.2014-0310 25848804

[B9] Battazza R. A. Suzuki F. S. Kalytczak M. M. Paunksnis M. R. Politi F. Evangelista A. L. (2019). Effects of previous carbohydrate supplementation on muscular fatigue: double-blind, randomized, placebo-controlled crossover study. *Motriz Revista Educação Física* 25:e101844.

[B10] Behrens M. Mau-Moeller A. Bruhn S. (2012). Effect of exercise-induced muscle damage on neuromuscular function of the quadriceps muscle. *Int. J. Sports Med.* 33 600–606. 10.1055/s-0032-1304642 22510801

[B11] Boccia G. Dardanello D. Brustio P. R. Tarperi C. Festa L. Zoppirolli C. (2018a). Neuromuscular fatigue does not impair the rate of force development in ballistic contractions of submaximal amplitudes. *Front. Physiol.* 9:1503. 10.3389/fphys.2018.01503 30405448 PMC6207600

[B12] Boccia G. Dardanello D. Tarperi C. Festa L. La Torre A. Pellegrini B. (2017a). Fatigue-induced dissociation between rate of force development and maximal force across repeated rapid contractions. *Hum. Mov. Sci.* 54 267–275. 10.1016/j.humov.2017.05.016 28595134

[B13] Boccia G. Dardanello D. Tarperi C. Festa L. La Torre A. Pellegrini B. (2018b). Women show similar central and peripheral fatigue to men after half-marathon. *Eur. J. Sport Sci.* 18 695–704. 10.1080/17461391.2018.1442500 29490592

[B14] Boccia G. Dardanello D. Zoppirolli C. Bortolan L. Cescon C. Schneebeli A. (2017b). Central and peripheral fatigue in knee and elbow extensor muscles after a long-distance cross-country ski race. *Scand. J. Med. Sci. Sports* 27 945–955. 10.1111/sms.12718 27293016

[B15] Brandon R. Howatson G. Strachan F. Hunter A. M. (2015). Neuromuscular response differences to power vs strength back squat exercise in elite athletes. *Scand. J. Med. Sci. Sports* 25 630–639. 10.1111/sms.12289 24995719

[B16] Buckthorpe M. Pain M. T. Folland J. P. (2014). Central fatigue contributes to the greater reductions in explosive than maximal strength with high-intensity fatigue. *Exp. Physiol.* 99 964–973. 10.1113/expphysiol.2013.075614 24728678

[B17] Buckthorpe M. Roi G. S. (2017). The time has come to incorporate a greater focus on rate of force development training in the sports injury rehabilitation process. *Muscles Ligaments Tendons J.* 7 435–441. 10.11138/mltj/2017.7.3.435 29387636 PMC5774916

[B18] Buckthorpe M. W. Hannah R. Pain T. Folland J. P. (2012). Reliability of neuromuscular measurements during explosive isometric contractions, with special reference to electromyography normalization techniques. *Muscle Nerve* 46 566–576. 10.1002/mus.23322 22987699

[B19] Cadore E. L. González-Izal M. Grazioli R. Setuain I. Pinto R. S. Izquierdo M. (2018). Effects of concentric and eccentric strength training on fatigue induced by concentric and eccentric exercise. *Int. J. Sports Physiol. Perform.* 14 91–98. 10.1123/ijspp.2018-0254 30204507

[B20] Cadore E. L. Pinheiro E. Izquierdo M. Correa C. S. Radaelli R. Martins J. B. (2013). Neuromuscular, hormonal, and metabolic responses to different plyometric training volumes in rugby players. *J. Strength Cond. Res.* 27 3001–3010. 10.1519/jsc.0b013e31828c32de 23442289

[B21] Cerqueira M. S. Pereira R. Mesquita G. N. D. Rocha T. Moura Filho A. G. D. (2019). Rate of force development to evaluate the neuromuscular fatigue and recovery after an intermittent isometric handgrip task with different blood flow restriction conditions. *Motriz Revista Educação Física* 25 1–6.

[B22] Chartogne M. Rahmani A. Nicolon L. Jubeau M. Morel B. (2020). Neuromuscular fatigability amplitude and aetiology are interrelated across muscles. *Exp. Physiol.* 105 1758–1766. 10.1113/ep088682 32822076

[B23] Conceição M. Cadore E. L. González-Izal M. Izquierdo M. Liedtke G. V. Wilhelm E. N. (2014). Strength training prior to endurance exercise: impact on the neuromuscular system, endurance performance and cardiorespiratory responses. *J. Hum. Kinet.* 44 171–181. 10.2478/hukin-2014-0123 25713678 PMC4327368

[B24] Conchola E. C. Thiele R. M. Palmer T. B. Smith D. B. Thompson B. J. (2015). Acute postexercise time course responses of hypertrophic vs. Power-endurance squat exercise protocols on maximal and rapid torque of the knee extensors. *J. Strength Cond. Res.* 29 1285–1294. 10.1519/jsc.0000000000000692 25774625

[B25] Cossich V. Maffiuletti N. (2020). Early vs. late rate of torque development: relation with maximal strength and influencing factors. *J. Electromyogr. Kinesiol.* 55:102486. 10.1016/j.jelekin.2020.102486 33152680

[B26] de Boer M. D. Maganaris C. N. Seynnes O. R. Rennie M. J. Narici M. V. (2007). Time course of muscular, neural and tendinous adaptations to 23 day unilateral lower-limb suspension in young men. *J. Physiol.* 583 1079–1091. 10.1113/jphysiol.2007.135392 17656438 PMC2277190

[B27] Del Vecchio A. Negro F. Holobar A. Casolo A. Folland J. P. Felici F. (2019). You are as fast as your motor neurons: speed of recruitment and maximal discharge of motor neurons determine the maximal rate of force development in humans. *J. Physiol.* 597 2445–2456. 10.1113/jp277396 30768687 PMC6487919

[B28] Dorneles J. R. Neto F. R. Gonçalves C. W. Costa R. R. G. Carregaro R. L. (2020). Does prolonged walking cause greater muscle fatigability in individuals with incomplete spinal cord injury compared with matched-controls? *Gait Posture* 78 65–71. 10.1016/j.gaitpost.2020.03.014 32268249

[B29] Drake D. Kennedy R. A. Wallace E. S. (2019). Measuring what matters in isometric multi-joint rate of force development. *J. Sport Sci.* 37 2667–2675. 10.1080/02640414.2019.1654595 31418319

[B30] Ereline J. Gapeyeva H. Pasuke M. (2004). Contractile changes in knee extensor muscles after repetitive maximal isokinetic contractions in male power-lifters and untrained subjects. *Medicina Dello Sport* 57 29–40.

[B31] Ewing J. L. Jr. Stull G. A. (1984). Rate of force development in the handgripping muscles by females as a function of fatigue level. *Res. Quart. Exerc. Sport* 55 17–23. 10.1080/02701367.1984.10605350

[B32] Farney T. M. Maclellan M. J. Hearon C. M. Johannsen N. M. Nelson A. G. (2018). The effect of aspartate and sodium bicarbonate supplementation on muscle contractile properties among trained men. *J. Strength Cond. Res.* 34 763–770. 10.1519/jsc.0000000000002692 30095737

[B33] Folland J. P. Buckthorpe M. W. Hannah R. (2014). Human capacity for explosive force production: neural and contractile determinants. *Scand. J. Med. Sci. Sports* 24 894–906. 10.1111/sms.12131 25754620

[B34] Gandevia S. C. (2001). Spinal and supraspinal factors in human muscle fatigue. *Physiol. Rev.* 81 1725–1789. 10.1152/physrev.2001.81.4.1725 11581501

[B35] Girard O. Bishop D. J. Racinais S. (2013). Hot conditions improve power output during repeated cycling sprints without modifying neuromuscular fatigue characteristics. *Eur. J. Appl. Physiol.* 113 359–369. 10.1007/s00421-012-2444-3 22743981

[B36] Girard O. Brocherie F. Millet G. P. (2016). High altitude increases alteration in maximal torque but not in rapid torque development in knee extensors after repeated treadmill sprinting. *Front. Physiol.* 7:97. 10.3389/fphys.2016.00097 27014095 PMC4789550

[B37] Girard O. Nybo L. Mohr M. Racinais S. (2015). Plantar flexor neuromuscular adjustments following match-play football in hot and cool conditions. *Scand. J. Med. Sci. Sports* 25 Suppl 1 154–163. 10.1111/sms.12371 25943666

[B38] Girard O. Racinais S. Périard J. D. (2014). Tennis in hot and cool conditions decreases the rapid muscle torque production capacity of the knee extensors but not of the plantar flexors. *Br. J. Sports Med.* 48 Suppl 1 i52–i58. 24668381 10.1136/bjsports-2013-093286 PMC3995226

[B39] Gordon J. A. III Hoffman J. R. Arroyo E. Varanoske A. N. Coker N. A. Gepner Y. (2017). Comparisons in the recovery response from resistance exercise between young and middle-aged men. *J. Strength Cond. Res.* 31 3454–3462. 10.1519/jsc.0000000000002219 28859014

[B40] Grazioli R. Lopez P. Andersen L. L. Machado C. L. F. Pinto M. D. Cadore E. L. (2019). Hamstring rate of torque development is more affected than maximal voluntary contraction after a professional soccer match. *Eur. J. Sport Sci.* 19 1336–1341. 10.1080/17461391.2019.1620863 31099729

[B41] Greco C. C. Da Silva W. L. Camarda S. R. Denadai B. S. (2013). Fatigue and rapid hamstring/quadriceps force capacity in professional soccer players. *Clin. Physiol. Funct. Imaging* 33 18–23. 10.1111/j.1475-097x.2012.01160.x 23216761

[B42] Gruber M. Gollhofer A. (2004). Impact of sensorimotor training on the rate of force development and neural activation. *Eur. J. Appl. Physiol.* 92 98–105. 10.1007/s00421-004-1080-y 15024669

[B43] Haff G. G. Ruben R. P. Lider J. Twine C. Cormie P. (2015). A comparison of methods for determining the rate of force development during isometric midthigh clean pulls. *J. Strength Cond. Res.* 29 386–395. 10.1519/jsc.0000000000000705 25259470

[B44] Häkkinen K. Myllylä E. (1990). Acute effects of muscle fatigue and recovery on force production and relaxation in endurance, power and strength athletes. *J. Sports Med. Phys. Fitness* 30 5–12. 2195236

[B45] Hatzikotoulas K. Patikas D. Ratel S. Bassa E. Kotzamanidis C. (2014). Central and peripheral fatigability in boys and men during maximal contraction. *Med. Sci. Sports Exerc.* 46 1326–1333. 10.1249/mss.0000000000000239 24389527

[B46] Jenkins N. D. M. Housh T. J. Traylor D. A. Cochrane K. C. Bergstrom H. C. Lewis R. W. (2014). The rate of torque development: a unique, non-invasive indicator of eccentric-induced muscle damage? *Int. J. Sports Med.* 35 1190–1195. 10.1055/s-0034-1375696 25259592

[B47] Jones D. A. (1996). High-and low-frequency fatigue revisited. *Acta Physiol. Scand.* 156 265–270. 10.1046/j.1365-201x.1996.192000.x 8729686

[B48] Kearney J. T. Stull G. A. (1981). Effect of fatigue level on rate of force development by the grip-flexor muscles. *Med. Sci. Sports Exerc.* 13 339–342. 7321834

[B49] Kelly L. A. Girard O. Racinais S. (2011). Effect of orthoses on changes in neuromuscular control and aerobic cost of a 1-h run. *Med. Sci. Sports Exerc.* 43 2335–2343. 10.1249/mss.0b013e31822037ca 21552159

[B50] King G. W. Stylianou A. P. Kluding P. M. Jernigan S. D. Luchies C. W. (2012). Effects of age and localized muscle fatigue on ankle plantar flexor torque development. *J. Geriatr. Phys. Ther.* 35 8–14. 10.1519/jpt.0b013e318221f53b 22189949

[B51] Krüger R. L. Aboodarda S. J. Jaimes L. M. Macintosh B. R. Samozino P. Millet G. Y. (2019). Fatigue and recovery measured with dynamic properties versus isometric force: effects of exercise intensity. *J. Exp. Biol.* 222(Pt 9):jeb197483. 10.1242/jeb.197483 30890621

[B52] Lanning A. C. Power G. A. Christie A. D. Dalton B. H. (2017). Influence of sex on performance fatigability of the plantar flexors following repeated maximal dynamic shortening contractions. *Appl. Physiol. Nutr. Metab.* 42 1118–1121. 10.1139/apnm-2017-0013 28636840

[B53] Lapole T. Ahmaidi S. Gaillien B. Leprêtre P. M. (2013). Influence of dorsiflexion shoes on neuromuscular fatigue of the plantar flexors after combined tapping-jumping exercises in volleyball players. *J. Strength Cond. Res.* 27 2025–2033. 10.1519/jsc.0b013e3182773271 23085976

[B54] Linnamo V. Hakkinen K. Komi P. V. (1998). Neuromuscular fatigue and recovery in maximal compared to explosive strength loading. *Eur. J. Appl. Physiol.* 77 176–181. 10.1007/s004210050317 9459539

[B55] Maffiuletti N. A. Aagaard P. Blazevich A. J. Folland J. Tillin N. Duchateau J. (2016). Rate of force development: physiological and methodological considerations. *Eur. J. Appl. Physiol.* 116 1091–1116. 10.1007/s00421-016-3346-6 26941023 PMC4875063

[B56] Marshall P. W. Finn H. T. Siegler J. C. (2015). The magnitude of peripheral muscle fatigue induced by high and low intensity single-joint exercise does not lead to central motor output reductions in resistance trained men. *PLoS One* 10:e0140108. 10.1371/journal.pone.0140108 26439261 PMC4595208

[B57] Marshall P. W. Lovell R. Jeppesen G. K. Andersen K. Siegler J. C. (2014). Hamstring muscle fatigue and central motor output during a simulated soccer match. *PLoS One* 9:e102753. 10.1371/journal.pone.0102753 25047547 PMC4105441

[B58] Marshall P. W. Metcalf E. Hagstrom A. D. Cross R. Siegler J. C. Enoka R. M. (2020). Changes in fatigue are the same for trained men and women after resistance exercise. *Med. Sci. Sports Exerc.* 52 196–204. 10.1249/mss.0000000000002103 31343516

[B59] Marshall P. W. Robbins D. A. Wrightson A. W. Siegler J. C. (2012). Acute neuromuscular and fatigue responses to the rest-pause method. *J. Sci. Med. Sport* 15 153–158. 10.1016/j.jsams.2011.08.003 21940213

[B60] Marshall P. W. M. Cross R. Haynes M. (2018). The fatigue of a full body resistance exercise session in trained men. *J. Sci. Med. Sport* 21 422–426. 10.1016/j.jsams.2017.06.020 28716692

[B61] McCaulley G. O. Mcbride J. M. Cormie P. Hudson M. B. Nuzzo J. L. Quindry J. C. (2009). Acute hormonal and neuromuscular responses to hypertrophy, strength and power type resistance exercise. *Eur. J. Appl. Physiol.* 105 695–704. 10.1007/s00421-008-0951-z 19066934

[B62] McLellan C. P. Lovell D. I. Gass G. C. (2011). The role of rate of force development on vertical jump performance. *J. Strength Cond. Res.* 25 379–385. 10.1519/jsc.0b013e3181be305c 20093963

[B63] Metcalf E. Hagstrom A. D. Marshall P. W. (2019). Trained females exhibit less fatigability than trained males after a heavy knee extensor resistance exercise session. *Eur. J. Appl. Physiol.* 119 181–190. 10.1007/s00421-018-4013-x 30324418

[B64] Millet G. Y. Lepers R. (2004). Alterations of neuromuscular function after prolonged running, cycling and skiing exercises. *Sports Med.* 34 105–116. 10.2165/00007256-200434020-00004 14965189

[B65] Minshull C. James L. (2013). The effects of hypohydration and fatigue on neuromuscular activation performance. *Appl. Physiol. Nutr. Metab.* 38 21–26. 10.1139/apnm-2012-0189 23368824

[B66] Moreira P. V. Gonçalves M. Crozara L. F. Castro A. Neto A. F. A. Goethel M. F. (2015). Effects of fatigue on the neuromuscular capacity of professional soccer players. *Isok. Exerc. Sci.* 23 275–282. 10.3233/ies-150588

[B67] Morel B. Rouffet D. M. Saboul D. Rota S. Clémençon M. Hautier C. A. (2015). Peak torque and rate of torque development influence on repeated maximal exercise performance: contractile and neural contributions. *PLoS One* 10:e0119719. 10.1371/journal.pone.0119719 25901576 PMC4406491

[B68] Nicholson G. Mcloughlin G. Bissas A. Ispoglou T. (2014). Do the acute biochemical and neuromuscular responses justify the classification of strength- and hypertrophy-type resistance exercise? *J. Strength Cond. Res.* 28 3188–3199. 10.1519/jsc.0000000000000519 24832969

[B69] Oliveira A. S. Caputo F. Aagaard P. Corvino R. B. Gonçalves M. Denadai B. S. (2013). Isokinetic eccentric resistance training prevents loss in mechanical muscle function after running. *Eur. J. Appl. Physiol.* 113 2301–2311. 10.1007/s00421-013-2660-5 23680937

[B70] Orssatto L. B. R. Moura B. M. Bezerra E. S. Andersen L. L. Oliveira S. N. Diefenthaeler F. (2018). Influence of strength training intensity on subsequent recovery in elderly. *Exp. Gerontol.* 106 232–239. 10.1016/j.exger.2018.03.011 29540305

[B71] Patrizio F. Ditroilo M. Felici F. Duranti G. De Vito G. Sabatini S. (2018). The acute effect of Quercetin on muscle performance following a single resistance training session. *Eur. J. Appl. Physiol.* 118 1021–1031. 10.1007/s00421-018-3834-y 29511920

[B72] Peñailillo L. Blazevich A. Numazawa H. Nosaka K. (2015). Rate of force development as a measure of muscle damage. *Scand. J. Med. Sci. Sports* 25 417–427. 10.1111/sms.12241 24798498

[B73] Power G. A. Dalton B. H. Rice C. L. Vandervoort A. A. (2013). Peak power is reduced following lengthening contractions despite a maintenance of shortening velocity. *Appl. Physiol. Nutr. Metab.* 38 1196–1205. 10.1139/apnm-2013-0092 24195619

[B74] Ravier G. Bouzigon R. Beliard S. Tordi N. Grappe F. (2018). Benefits of compression garments worn during handball-specific circuit on short-term fatigue in professional players. *J. Strength Cond. Res.* 32 3519–3527. 10.1519/jsc.0000000000001342 26840438

[B75] Rice D. A. Mannion J. Lewis G. N. Mcnair P. J. Fort L. (2019). Experimental knee pain impairs joint torque and rate of force development in isometric and isokinetic muscle activation. *Eur. J. Appl. Physiol.* 119 2065–2073. 10.1007/s00421-019-04195-6 31332518

[B76] Rissanen J. A. Häkkinen A. Laukkanen J. Kraemer W. J. Häkkinen K. (2020). Acute neuromuscular and hormonal responses to different exercise loadings followed by a sauna. *J. Strength Cond. Res.* 34 313–322. 10.1519/jsc.0000000000003371 31490429

[B77] Rodriguez-Rosell D. Pareja-Blanco F. Aagaard P. Gonzalez-Badillo J. J. (2017). Physiological and methodological aspects of rate of force development assessment in human skeletal muscle. *Clin. Physiol. Funct. Imaging* 38 743–762. 10.1111/cpf.12495 29266685

[B78] Siegler J. C. Marshall P. W. Raftry S. Brooks C. Dowswell B. Romero R. (2013). The differential effect of metabolic alkalosis on maximum force and rate of force development during repeated, high-intensity cycling. *J. Appl. Physiol.* 115 1634–1640. 10.1152/japplphysiol.00688.2013 24092691

[B79] Storey A. Wong S. Smith H. K. Marshall P. (2012). Divergent muscle functional and architectural responses to two successive high intensity resistance exercise sessions in competitive weightlifters and resistance trained adults. *Eur. J. Appl. Physiol.* 112 3629–3639. 10.1007/s00421-012-2346-4 22350356

[B80] Strojnik V. Komi P. V. (2000). Fatigue after submaximal intensive stretch-shortening cycle exercise. *Med. Sci. Sports Exerc.* 32 1314–1319. 10.1097/00005768-200007000-00020 10912899

[B81] Taipale R. S. Häkkinen K. (2013). Acute hormonal and force responses to combined strength and endurance loadings in men and women: the “order effect”. *PLoS One* 8:e55051. 10.1371/journal.pone.0055051 23408956 PMC3567118

[B82] Thompson B. J. Ryan E. D. Herda T. J. Costa P. B. Herda A. A. Cramer J. T. (2014). Age-related changes in the rate of muscle activation and rapid force characteristics. *Age* 36 839–849. 10.1007/s11357-013-9605-0 24338233 PMC4039274

[B83] Thorlund J. B. Aagaard P. Madsen K. (2009). Rapid muscle force capacity changes after soccer match play. *Int. J. Sports Med.* 30 273–278. 10.1055/s-0028-1104587 19199196

[B84] Thorlund J. B. Michalsik L. B. Madsen K. Aagaard P. (2008). Acute fatigue-induced changes in muscle mechanical properties and neuromuscular activity in elite handball players following a handball match. *Scand. J. Med. Sci. Sports* 18 462–472. 10.1111/j.1600-0838.2007.00710.x 18028284

[B85] Tillin N. A. Jimenez-Reyes P. Pain M. T. Folland J. P. (2010). Neuromuscular performance of explosive power athletes versus untrained individuals. *Med. Sci. Sports Exerc.* 42 781–790. 10.1249/mss.0b013e3181be9c7e 19952835

[B86] Tricco A. C. Lillie E. Zarin W. O’brien K. K. Colquhoun H. Levac D. (2018). PRISMA extension for scoping reviews (PRISMA-ScR): checklist and explanation. *Ann. Intern. Med.* 169 467–473. 30178033 10.7326/M18-0850

[B87] Váczi M. Tékus E. Kaj M. Kõszegi T. Ambrus M. Tollár J. (2013). Changes in metabolic and muscle damage indicators following a single bout of jump training on stair versus at level. *Acta Physiol. Hung.* 100 445–456. 10.1556/aphysiol.100.2013.010 24013940

[B88] Valkeinen H. Ylinen J. Malkia E. Alen M. Hakkinen K. (2002). Maximal force, force/time and activation/coactivation characteristics of the neck muscles in extension and flexion in healthy men and women at different ages. *Eur. J. Appl. Physiol.* 88 247–254. 10.1007/s00421-002-0709-y 12458368

[B89] Viitasalo J. T. Komi P. V. (1981). Effects of fatigue on isometric force- and relaxation-time characteristics in human muscle. *Acta Physiol. Scand.* 111 87–95. 10.1111/j.1748-1716.1981.tb06709.x 7223455

[B90] Vila-Chã C. Hassanlouei H. Farina D. Falla D. (2012). Eccentric exercise and delayed onset muscle soreness of the quadriceps induce adjustments in agonist-antagonist activity, which are dependent on the motor task. *Exp. Brain Res.* 216 385–395. 10.1007/s00221-011-2942-2 22094715 PMC3262141

[B91] Wallace J. W. Power G. A. Rice C. L. Dalton B. H. (2016). Time-dependent neuromuscular parameters in the plantar flexors support greater fatigability of old compared with younger males. *Exp. Gerontol.* 74 13–20. 10.1016/j.exger.2015.12.001 26657724

[B92] Zhou S. (1996). Acute effect of repeated maximal isometric contraction on electromechanical delay of knee extensor muscle. *J. Electromyogr. Kinesiol.* 6 117–127. 10.1016/1050-6411(95)00024-0 20719669

[B93] Zhou S. Carey M. F. Snow R. J. Lawson D. L. Morrison W. E. (1998). Effects of muscle fatigue and temperature on electromechanical delay. *Electromyogr. Clin. Neurophysiol.* 38 67–73. 9553743

[B94] Zhou S. Mckenna M. J. Lawson D. L. Morrison W. E. Fairweather I. (1996). Effects of fatigue and sprint training on electromechanical delay of knee extensor muscles. *Eur. J. Appl. Physiol.* 72 410–416. 10.1007/bf00242269 8925810

